# Targeted Amplicon Deep Sequencing for Monitoring Antimalarial Resistance Markers in Western Kenya

**DOI:** 10.1128/aac.01945-21

**Published:** 2022-03-10

**Authors:** Victor Osoti, Mercy Akinyi, Kevin Wamae, Kelvin M. Kimenyi, Zaydah de Laurent, Leonard Ndwiga, Paul Gichuki, Collins Okoyo, Stella Kepha, Charles Mwandawiro, Regina Kandie, Philip Bejon, Robert W. Snow, Lynette Isabella Ochola-Oyier

**Affiliations:** a KEMRI-Wellcome Trust Collaborative Programme, Kilifi, Kenya; b Institute of Primate Research, National Museums of Kenya, Nairobi, Kenya; c Department of Biochemistry, University of Nairobi, Nairobi, Kenya; d Eastern and Southern Africa Centre of International Parasite Control, Kenya Medical Research Institute, Nairobi, Kenya; e Division of National Malaria Programme, Ministry of Health, Nairobi, Kenya; f Centre for Tropical Medicine and Global Health, Nuffield Department of Medicine, University of Oxford, Oxford, United Kingdom

**Keywords:** Kenya, *Plasmodium falciparum*, antimalarial agents, deep sequencing, drug resistance

## Abstract

Molecular surveillance of Plasmodium falciparum parasites is important to track emerging and new mutations and trends in established mutations and should serve as an early warning system for antimalarial resistance. Dried blood spots were obtained from a Plasmodium falciparum malaria survey in school children conducted across eight counties in western Kenya in 2019. Real-time PCR identified 500 P. falciparum-positive samples that were amplified at five drug resistance loci for targeted amplicon deep sequencing (TADS). The absence of important kelch 13 mutations was similar to previous findings in Kenya pre-2019, and low-frequency mutations were observed in codons 569 and 578. The chloroquine resistance transporter gene codons 76 and 145 were wild type, indicating that the parasites were chloroquine and piperaquine sensitive, respectively. The multidrug resistance gene 1 haplotypes based on codons 86, 184, and 199 were predominantly present in mixed infections with haplotypes NYT and NFT, driven by the absence of chloroquine pressure and the use of lumefantrine, respectively. The sulfadoxine-pyrimethamine resistance profile was a “superresistant” combination of triple mutations in both *Pfdhfr* (51I 59R 108N) and *Pfdhps* (436H 437G 540E), rendering sulfadoxine-pyrimethamine ineffective. TADS highlighted the low-frequency variants, allowing the early identification of new mutations, *Pfmdr1* codon 199S and *Pfdhfr* codon 85I and emerging 164L mutations. The added value of TADS is its accuracy in identifying mixed-genotype infections and for high-throughput monitoring of antimalarial resistance markers.

## INTRODUCTION

Chloroquine and sulfadoxine-pyrimethamine (SP) resistance in Africa was historically imported from Southeast Asia (1, 2). Additionally, SP resistance has also developed independently at multiple sites in Africa (3). Recently, the indigenous detection of an artemisinin resistance mutation has been described in Rwanda, and a few African countries have reported mutations in *Plasmodium falciparum* kelch 13 (Pfk13), though at low frequencies (4, 5). Both scenarios of independent emergence and the global spread of resistance emphasize the need for regular surveillance of parasite resistance mutations. Genomic surveillance provides an important tool for drug resistance monitoring using routine blood sample collections from health facilities, community surveys, or therapeutic efficacy studies (TES). Uwimana et al. (4) conducted a molecular surveillance study, genotyping pretreatment samples from a TES at Pfk13, the artemisinin resistance-conferring gene. They identified an artemisinin resistance-associated mutation, R561H, and using whole-genome sequencing showed that the mutation arose independently in Rwanda. They later demonstrated through a TES the association between the R561H mutation and delayed parasite clearance; parasites containing this mutation were observed on day 3 (6). Thus, molecular surveillance is a rapid and powerful approach for the early detection and monitoring of known drug resistance mutations.

An easy route to implementing and establishing antimalarial resistance marker surveillance is by examining known genes such as the markers described below. Artemisinin resistance compromises the use of artemisinin-based combination therapies (ACTs) and selects for partner drug resistance (7, 8). Mutations in Pfk13, including Y493H, R539T, I543T, and C580Y, are strongly associated with artemisinin resistance, resulting in delayed parasite clearance (9). The Plasmodium falciparum chloroquine resistance transporter (*Pfcrt*) mutation K76T (10, 11) and multidrug resistance gene 1 (*Pfmdr1*) (12) mutations N86Y, Y184F, and D1246Y mediate resistance to drugs such as chloroquine and amodiaquine (aminoquinolines) (13). *Pfcrt* and *Pfmdr1* are also associated with decreased sensitivity to amodiaquine and lumefantrine (ACT partner drugs), but the effects of these polymorphisms on therapeutic responses to artesunate-amodiaquine and artemether-lumefantrine (AL) have not been clearly defined (14). Furthermore, *Pfmdr1* mutations have also been associated with resistance to mefloquine and artemisinin (15–17). Additional *Pfcrt* mutations, T93S, H97Y, F145I, and I218F, downstream of codon 76 were associated with dihydroartemisinin-piperaquine (DHA-PPQ) treatment failure in the Greater Mekong subregion, in Cambodia, Thailand, and Vietnam (18, 19). In addition, an association of a 5-fold-increased risk of DHA-PPQ treatment failure was observed with the F145I mutation (20).

Point mutations in the genes encoding dihydrofolate reductase (*Pfdhfr*) and dihydropteroate synthase (*Pfdhps*), the two key enzymes in the folate biosynthesis pathway, which mediate resistance to the antifolate drugs sulfadoxine and pyrimethamine, respectively, have been reported in numerous studies (21). The mutations in *Pfdhfr* arise in a stepwise manner, initially at codon S108N and thereafter in the following codons N51I, C59R, and I164L, in this order (22). Every additional mutation leads to a 1-fold change-increased level of resistance to pyrimethamine (23–25). These mutations, when combined with mutations in *Pfdhps*, result in the widespread quintuple mutation (*Pfdhfr* codons 51, 59, and 108, plus *Pfdhps* codons 437 and 540), termed a fully resistant combination, that is characteristic of East African parasites (26) and are good predictors of SP treatment failure in children (27, 28). An additional *Pfdhps* mutation at codon 581 has been linked to a high rate of therapeutic failure (29), and the prevalence of the triple mutant (A437G K540E A581G) was shown to increase in Tanzania (30).

In this study, these five well-described antimalarial resistance markers (31, 32) were examined in a survey of asymptomatic school children using a malaria rapid diagnostic test (RDT) in western Kenya. The aim was to determine the parasite drug resistance profile in the region as part of a scaled approach to monitoring resistance using the high-throughput next-generation sequencing (NGS) technique known as targeted amplicon deep sequencing (TADS).

## RESULTS

### Sample demographics.

Following the screening of 8,111 children aged 4 to 18 years attending 82 schools across the 8 counties of Western Kenya, 28% (*n* = 2,247) were malaria positive by RDT. The dried blood spots (DBSs) from RDT-positive children were screened by 18S rRNA reverse transcription-PCR (RT-PCR) to identify 1,263 (56%) samples with detectable levels of DNA to take forward for TADS. The median (range) RT-PCR cycle threshold (*C_T_*) for the samples was 31 (19 to 39). An initial random set of 192 samples were screened, and over 50% of the samples did not yield a PCR product for the genes. Therefore, to generate a feasible number of amplicons in duplicate for TADS, samples with low parasitemia above the median *C_T_* of 31 and those from schools with a low representation of positive samples (<10) were excluded. In total, 500 samples were used to generate amplicons in duplicate for *Pfdhfr*, *Pfdhps*, *Pfk13*, and *Pfmdr1* genes. Sequence data were obtained from 322, 225, 196, and 98 samples, respectively (Table 1). Additionally, the reference DNA isolates were mixed in different ratios, and the deep sequencing assay did detect haplotype frequencies at the lowest proportion of 1%, providing confidence in the detection of the low-frequency variants in the sample sequence data. As indicated in Materials and Methods, *Pfcrt* was not amplified, warranting the use of capillary sequencing to generate over 200 samples with sequence data (Table 1).

**TABLE 1:** Frequency of mutations from each drug resistant gene in the population

Gene	Mutation	No. successfully sequenced	Frequency (%)^*a*^		
			Wild-type	Mutant	Mixed
*crt*	K76T	203	100	0	NA
	F145I	369	100	0	NA
*dhfr*	N51I	322	0	98.4	1.6
	C59R	322	0.3	89.0	10.6
	T85I	322	99.1	0	0.9
	S108N	322	0	98.4	1.6
	I164L	322	87.9	0.3	11.8
*mdr1*	N86Y	98	100	0	0
	Y184F	98	16.3	8.2	75.5
	T199S	98	90.8	0	9.2
*dhps*	S436H	225	2	67	31
	A437G	225	0	100	0
	K540E	225	0	100	0
	A581G	225	98	0	2
*k13*	F446I	196	100	0	0
	N458Y	196	100	0	0
	M476I	196	100	0	0
	Y493H	196	100	0	0
	R539T	196	100	0	0
	I543T	196	100	0	0
	P553L	196	100	0	0
	R561H	196	100	0	0
	A569S	196	99	0	1
	A578S	196	92.3	0	7.7
	C580Y	196	100	0	0

a “Mixed” indicates infections with both the mutant and the wild-type allele. NA, not applicable, as this could not be accurately determined.

### Prevalence of mutations of aminoquinoline genetic markers.

Three SNP loci were genotyped in *Pfmdr1*, two of which were variant codons, leading to three haplotypes, including wild-type infections, which were the second most prevalent in the population (Table 2). The majority of parasite genotypes were mixed wild-type (i.e., NYT) and variant NFT, which indicates selection by lumefantrine. A newly described mutation was observed at codon 199 (Table 1) (33), leading to a new haplotype (NYS) that was rare and observed in mixed infections (9.1%). The *Pfcrt* data indicated that 100% of the parasites were wild type for both single nucleotide polymorphisms (SNPs) genotyped, and mixed infections could not be accurately determined using the capillary sequencing method and thus were not analyzed (Table 1).

**TABLE 2:** Frequency of resistance haplotypes for each gene and of infections containing mixed haplotypes

Gene	Resistance combination	Resistance phenotype^*a*^	Frequency [no. (%)]	Haplotype(s)^*b*^	Frequency [no. (%)]
*dhfr*	Quadruple mutant	Superresistant (presence of IRTNL)	43 (13.2)	ICTNI^d^, ICTNL^t^, IRTNI^t^, IRTNL^q^	3 (0.9)
				ICTNI^d^, IRTNI^t^, IRTNL^q^	4 (1.2)
				IRTNI^t^, IRTNL^q^	32 (9.9)
				IRTNI^t^, IRINI^q^	3 (0.9)
				IRTNL	1 (0.3)
	Triple mutant	Fully resistant (presence of IRTNI)	278 (86.5)	ICTNI^d^, IRTNI^t^	22 (7)
				IRTNI^t^	251 (78)
				NCTSI*, ICTNI^d^, IRTNI^t^	1 (0.3)
				NCTSI*, NCTNI^s^, IRTNI^t^	2 (0.6)
				NCTSI*, IRTNI^t^	2 (0.6)
	Double mutant	Partially resistant (ICTNI)	1 (0.3)	ICTNI^d^	1 (0.3)
	Wild type	Sensitive (NCTSI)	0	NA	0
*dhps*	Quadruple mutant	Superresistant (presence of HGEG)	4 (1.8)	SGEA^d^, HGEA^t^, HGEG^q^	2 (0.9)
				HGEA^t^, HGEG^q^	2 (0.9)
	Triple mutant	Fully resistant (presence of HGEA)	216 (96)	SGEA^d^, HGEA^t^	68 (30.2)
				HGEA^t^	148 (65.8)
	Double mutant	Fully resistant (SGEA)	5 (2.2)	SGEA^d^	5 (2.2)
	Wild type	Sensitive (SAKA)	0	NA	0
*k13*	Single mutant	NA	15 (7.7)	FNMYRIPRAAC*, FNMYRIPRASC	15 (7.7)
			2 (1)	FNMYRIPRAAC*, FNMYRIPRSAC	2 (1)
	Wild type	Sensitive (FNMYRIPRAAC)	179 (91.3)	FNMYRIPRAAC*	179 (91.3)
*mdr1*	Double mutant	NA	82 (83.7)	NFT^d^, NYS^t^	1 (1)
				NFT^d^	8 (8.2)
				NYT*, NFT^d^, NYS^d^	6 (6.1)
				NYT*, NFT	67 (68.4)
			2 (2)	NYT*, NYS^t^	2 (2)
	Wild type	Sensitive (NYT)	14 (14.3)	NYT*	14 (14.3)

a Resistance phenotypes are based on the *Pfdhfr* + *Pfdhps* mutation combinations. “super” relates to the triple + triple (sextuple) or quadruple + triple (septuple) combinations; “fully” relates to the triple + double (quintuple) combination; “partially” relates to the triple + single (quadruple) combination. The contribution of *Pfdhps* in the combinations is dependent on codons 437G, 540E, and 581G. NA, not applicable.

b Superscripts are as follows: *, wild type; d, double mutation; t, triple mutation; q, quadruple mutation.

### SP resistance markers.

Within *Pfdhfr*, five SNPs were genotyped. The mutation T85I is unique, since it has, to our knowledge, not been described before (Table 1). Codon 85 was primarily wild type, with only three infections harboring a mixed genotype (Table 1). The mutant allele was a low-frequency variant in each of the three individual infections, contributing 11%, 3%, and 1% of 281, 872, and 1,507 sequence reads, respectively. The 164L mutation is of growing concern, since it results in the quadruple mutant (51I 59R 108N 164L), which was rare in the study area and was mainly observed in mixed infections (12.3%). However, the interrogation of individual mixed-genotype infections indicated that the 164L mutation was the dominant allele or was present at nearly equal frequencies with the wild-type genotype. The common *Pfdhfr* triple mutant (51I 59R 108N) was also prevalent in the study population at 78%. All other haplotypes, including the wild type, were rare, though remarkably, 22% of the infections were classified as quadruple mutant based on the number of mixed-genotype infections containing the quadruple haplotype (Table 2). Four *Pfdhps* mutations were defined, with the inclusion of the recently described (34) 436H mutation. Though codons 437 and 540 were mutant in all (100%) the samples (Table 1), the common double-mutant haplotype was at a low frequency, 2.2%. However, the triple mutant haplotype, a combination of the 436H mutant and the double mutant, was prevalent, while the quadruple mutant which included the 581G mutation was rare (Table 2).

### Artemisinin resistance marker.

All the validated *Pfk13* artemisinin resistance codons (446, 458, 476, 493, 539, 543, 553, 561, and 580) were 100% wild type. The mutations A569S, previously described in Uganda at <1% frequency (35, 36), and A578S (which is rare but consistently observed in Kenya [32, 34, 37] and across Africa [38, 39]) were observed at low frequencies as mixed-genotype infections at 1% and 8%, respectively (Table 1). Only 3 haplotypes were therefore observed, and the wild type was dominant at a frequency of 91.3% (Table 2).

## DISCUSSION

TADS provided a rapid, high-throughput, and detailed analysis of well-described drug resistance markers, robustly detecting low-frequency variants and quantifying the proportion of variants of each gene per sample to allow the classification of sensitive (wild-type), resistant (mutant), or mixed-genotype infections. Newly defined rare variants (*Pfmdr1* codon 199 and *Pfdhfr* codon 85I and 164L mutations) should be surveyed temporally to monitor a rise in frequency and evidence of directional selection. TADS improved the detection of *Pfdhfr* codon 164, to identify quadruple mutants (and the “superresistance” *dhfr-dhps*) combination (26), whose frequencies may have recently been underestimated, at a frequency of 4%, by capillary sequencing (34). Importantly, TADS underscored the genetic complexity of drug resistance previously unquantifiable by genotyping methods such as capillary sequencing, with some infections comprising up to four mutant haplotypes.

The *Pfdhfr* 164L mutation was observed once in Kilifi between 2006 and 2008, showing high *in vitro* levels of resistance to pyrimethamine (40). Although the 164L mutation is still rare in Kenya and there was no detection of this mutation in neighboring Tanzania (41), its frequency was ∼80% in some regions of Uganda in 2019 (42). Notably, the *Pfdhfr* triple mutant (codons 51I, 59R, and 108N) was thought to be at fixation due to the absence of sensitive (NCS) parasites between 2006 and 2013 in symptomatic children in the coastal region of Kenya, where malaria is endemic (31). However, TADS detected the presence of the sensitive genotype in the mixed infections.

The *Pfdhps* double mutant has reached fixation, while the 581G mutation is rare in the study population. The low frequency of the 581G mutation has also been described before in the study area, Siaya County, at a prevalence of 3% in 2017-2018 (34) and at 1.1% in Tanzania in 2019 (41). Once again, in comparison to some regions of Uganda, 581G was at a frequency of 40% (42). The newly described *Pfdhps* 436H mutation appears to be unique to Kenya, with no recent data describing this mutation in Uganda (42), Tanzania (41), or Sudan (43). It seems to have taken over from the 436A mutation (detected in 2010) in Siaya (34), and a low prevalence (<3%) was detected only in 1998-1999 in Kilifi (32).

Recent data from Uganda of a septuple mutant, a combination of *Pfdhfr* quadruple mutant (51I 59R 108N 164L) and *Pfdhps* triple mutant (437G 540E 581G), suggest that these superresistant combination parasites will escape SP treatment; however, SP in intermittent preventive treatment for malaria in pregnancy (IPTp) remains efficacious (42). In Tanzania, parasites were contrasting and primarily quintuple mutants with a fully resistant combination (*dhfr* 59R 108N and *dhps* 437G 540E) (41). This same *dhfr*-*dhps* combination was observed in Ethiopia, which is unique because there is no IPTp-SP, and hence, there was a reduction in the quintuple frequency between 2005 and 2008 (44). In contrast, in Sudan, the quadruple mutant (*dhfr* 51I 108N and *dhps* 437G 540E) dominated (43). The profile in western Kenya was also distinct, a sextuple mutation (superresistant) combination of *Pfdhfr* (51I 59R 108N) and *Pfdhps* (436H 437G 540E) triple mutants, which still renders SP ineffective. Similar to mutations found in Uganda and Tanzania, these mutations do not appear to have a clear impact on the efficacy of SP in IPTp, since IPTp-SP is thought to be effective in clearing parasites in Kenya (45). The great contrast in mutation combinations is potentially due to differences in malaria transmission intensity, which would lead to differences in the extent of antimalarial drug use and selective pressure.

Interestingly, the *dhfr* and *dhps* quintuple-, sextuple-, and septuple-mutant parasites confer resistance to SP, making the drug ineffective for treatment. However, SP in IPTp continues to be effective, despite the widespread and high prevalence of mutant parasites. Thus, the mechanism through which SP is effective as a chemoprophylactic drug is not well understood. It may be due to the long half-life of the drug combination of 4 to 7 days (46, 47) circulating in the bloodstream in advance of an infection, thus minimizing a rapid increase in the resistant parasite biomass and symptomatic malaria. Additional studies are required to determine the benefits and effectiveness of SP use; simple studies of its use among pregnant women and as a possible chemopreventative strategy in infants are required to understand the modulatory mechanisms that determine the efficacy of SP amid the molecular resistance.

As expected, there were no *Pfk13* artemisinin resistance-conferring mutations identified, and the parasites were entirely sensitive to chloroquine and piperaquine. The complete shift to a chloroquine-sensitive population is consistent with previous trends in coastal (32) and western (48) Kenya. This finding provokes the debate regarding a reintroduction of the previously very successful drug for treatment, most likely in a combination therapy. However, its known side effects and toxicity (49) minimize the possibility of its use.

*Pfmdr1* also follows the earlier trends observed in coastal (32) and western Kenya (48) of a complete reversion to the N86 sensitive genotype, driven by the cessation of chloroquine use in Kenya from the late 1990s due to *mdr1* being associated with resistance to chloroquine (50). In contrast, the high prevalence of the NF haplotype in mixed infections has previously been attributed to lumefantrine drug pressure (51, 52). The evidence from *in vivo* susceptibility analyses of lumefantrine revealed that the 86N 184F 1246D haplotype gave rise to parasites tolerating a 15-fold-higher concentration of lumefantrine than the parasites containing the triple-mutant YYY haplotype (53).

The use of RDTs in this study provided a quick and scalable screening tool for identifying malaria-positive samples from asymptomatic school-aged children. They are, however, limited in their sensitivity, since the histidine-rich protein 2 marker assessed by the RDT can linger in the bloodstream long after individuals clear parasites (54, 55). Therefore, real-time, quantitative PCR was a quick way of identifying samples for downstream analysis on NGS platforms, such as TADS. Slightly more than half the RDT-positive samples were RT-PCR positive, indicating an overestimation of the number of malaria-positive asymptomatic individuals. Nested PCR increased the detection of low-parasite-density infections. However, it was also a rate-limiting step that was mitigated by combining the PCR amplicons from all 4 genes into one NGS run, increasing the throughput of genotyped amplicons. A potential improvement of the process is running single amplicons per sample rather than duplicates, because the mutant codons are well described. Additionally, the *C_T_* cutoff of <31 reduced the sample size substantially, and an extension to a *C_T_* cutoff of <39 is more appropriate due to the inclusion of the nested-PCR step.

Antimalarial resistance marker monitoring continues to be a high-priority activity in Kenya and the region, and tools such as TADS provide a scalable method for routine molecular surveillance that rapidly generates data on the distribution and extent of spread of resistance and any new emerging variants for policy decision making.

## MATERIALS AND METHODS

### Study area and sampling.

Kenya supports a diverse range of malaria transmission ecologies (56). The most intense, perennial transmission continues to occur in the densely populated eight counties surrounding Lake Victoria in western Kenya. Since 2010, these counties have been the focus of decentralized, subnational intensified vector control, intermittent presumptive treatment of malaria in pregnancy, improved diagnosis, artemisinin-based combination therapy for case management, and, in September 2019, the pilot introduction of the RTS,S vaccine (57).

The Kenya national malaria control program and the Kenya Medical Research Institute (KEMRI) have maintained school-based malaria infection surveys since 2009 to track changes in transmission intensity (58–61). Between February and March 2019, these surveys were repeated across the eight counties of western Kenya. In calculating the sample size, a stratified sampling frame of all public day schools was used to randomly select one school per subcounty administrative unit, ensuring a minimum of 10 schools per county, allowing adequate precision in the county-level estimates of P. falciparum infection prevalence based upon the county predicted infection prevalence in 2015 (56) and a presumed design effect of clustering between schools of two, derived from previous school surveys (58). At each selected school, 10 boys and 10 girls were randomly selected from classes 2 to 6 to provide ca. 100 children per school.

Trained interviewers asked each participating child about details related to their age, bed net use, and any illness on the day of the survey. Each child was asked to provide a finger-prick blood sample for a rapid diagnostic test (RDT) (CareStart) and an ∼50-μL dried blood spot (DBS) on Whatman 3-mm filter paper (Sigma). The DBS samples were allowed to air dry for at least 1 h and individually packed in zip-lock bags, with a desiccant, to prevent cross-contamination and delivered in a cooler box to the nearest health care facility for short-term storage, before shipment to the KEMRI-Wellcome Trust Research Programme laboratories.

The study protocol received ethical approval from the KEMRI and National Ethics Review Committee (number KEMRI/SERU/ESACIPAC/11/3822). Additional approval was provided by the appropriate county-level health and education authorities, who were briefed about the survey. At the school level, parental consent was based on passive, opt-out consent rather than written opt-in consent owing to the low risk and routine nature of the study procedures (62). Individual assent was obtained from each child before participation in the survey. All children with a malaria-positive RDT were treated with artemether-lumefantrine according to the national malaria treatment guidelines, and written advice on subsequent doses was provided to the child and class teacher.

### Parasite DNA extraction.

Parasite DNA was extracted from two areas of the ∼50-μL DBS using the Chelex-saponin method (63). Each DBS was punched (two 2.5-mm discs) with a sterile (absolute ethanol [>96%] and a flame) puncher at the center and periphery and transferred to a 2-mL 96-well master block plate with sterile tweezers. The samples were lysed overnight using 1 mL of 0.5% (wt/vol) saponin in 1× phosphate-buffered saline (PBS). Following saponin aspiration, the discs were incubated in 1 mL 1× PBS at 4°C for 30 min; thereafter, 150 μL of a solution of 6% (wt/vol) Chelex in DNase/RNase-free water was used to incubate the samples for 30 min at 97°C. At regular (10-min) intervals, the samples were vortexed and centrifuged to maximize the elution of DNA. The plates were then centrifuged at 4,000 × *g* for 5 min, and 120 μL of the DNA-containing solution was stored at −20°C for further analyses.

### P. falciparum qPCR.

The parasite DNA was amplified using the TaqMan probe on the ABI Prism 7500 HT real-time system (Applied Biosystems) following a previously described method (64). The TaqMan probe-based PCR amplified a 133-bp amplicon from the multicopy (3 per parasite) 18S (small-subunit) rRNA gene (GenBank accession number M19173) (see Table S1 in the supplemental material). A 25-µL final quantitative PCR (qPCR) volume was prepared as follows: 2.5 µL each of P. falciparum 18S rRNA forward and reverse primers (10 pmol/µL), 0.625 µL of 18S minor groove binder (MGB) probe (10 pmol/µL), 12.5 µL 2× TaqMan universal PCR master mix, 6.75 µL of sample or 3D7 control samples, with the remaining volume PCR clean water. The reaction was done under the following qPCR cycling conditions: 50°C for 2 min (AmpErase step), 95°C for 10 min (hot-start activation), and then 45 cycles of 95°C for 15 s (denaturing) and 95°C for 1 min (annealing and extension). Eight sequencing controls were also prepared from P. falciparum laboratory reference isolates (3D7, HB3, and 7G8) (BEI Resources) and DNA representing different apical membrane antigen 1 (*ama1*) variants. The reference DNA isolates were mixed in proportions of 1:1:1, 1:0:0, 0:0.5:0.5, 0:0:1, 0.5:0.25:0.25, 0.44:0.33:0.22, 0.5:0.45:0.05, and 0.5:0.49:0.01 to determine the lowest limit of variant detection in the assay.

### Drug resistance marker genotyping.

The drug resistance marker genes *Pfcrt* (PF3D7_0709000), *Pfdhfr* (PF3D7_0417200), *Pfdhps* (PF3D7_0810800), *Pfk13* (PF3D7_1347700), and *Pfmdr1* (PF3D7_0523000) and *ama1* (PF3D7_1133400) from the DNA reference isolates were amplified using nested PCR. The first PCR assay was set up as a 10-µL final reaction volume as follows: 1 µL of template DNA (<50 ng), 0.14 µL Expand high-fidelity DNA polymerase (3.5 U/µL) (Roche, USA), 0.3 µL forward and reverse 10 mM external primers (Table S1), 0.2 µL of 10 µM deoxynucleoside triphosphates (dNTPs) (Bioline), 1 µL each of buffers 2 and 4, and 6.56 µL of nuclease-free water. The nested PCR was prepared as described above, except that 1 µL of the first PCR assay product with multiplex identifier (MID) (Roche, USA)-tagged (Table S2) internal forward primers and untagged reverse internal primers (Table S1) were used. Each sample was amplified in duplicate with nonoverlapping MID tags. The following PCR cycling conditions were used: 94°C for 2 min, 10 cycles of 94°C for 15 s, 52°C for 30 s, and 72°C for 45 s, followed by an additional 20 cycles of 94°C for 15 s, 52°C for 30 s, and 72°C for 45 s and a final elongation step of 72°C for 5 min. Successful PCR amplification was confirmed using 1% (wt/vol) agarose gels stained with RedSafe nucleic acid staining solution (iNtRON Biotechnology DR).

### Amplicon library preparation and sequencing.

PCR amplicons were mixed to create amplicon pools of nonoverlapping MIDs. Since 16 unique MIDs were used, this allowed up to 8 samples per gene to be sequenced in duplicate in each sequencing library. The first sequencing run included 59 *Pfmdr1* samples in duplicate and 22 samples without duplicates (14 pools), 314 *Pfk13* duplicate samples and 98 samples without duplicates (79 pools), and the 8 *ama1* control mixtures. These amplified products were purified using the Zymo ZR-96 DNA Clean & Concentrator-5 kit (Zymo Research) according to the manufacturer’s instructions, and products were then eluted in 30 µL of DNA elution buffer. In contrast, for the second run, the following amplicons were included: 83 samples in duplicate (18 pools) for *Pfmdr1*,  386 samples in duplicate (50 pools) for *Pfdhfr*, 422 samples in duplicate (59 pools) for *Pfdhps*, and the 8 *ama1* control mixtures. These amplicons were purified using AMPure XP SPRI beads (Beckman Coulter, Inc.) as per the manufacturer’s instructions and eluted in 20 μL of PCR-grade water. Purified DNA from the two sequencing runs was quantified using a Qubit double-strand DNA (dsDNA) high-sensitivity (HS) assay kit (Invitrogen) according to the manufacturer’s instructions. Thereafter, the PCR amplicons were normalized to equal amounts of 1 ng each using EB buffer (Qiagen) followed by library construction. Library preparation was done using the Kapa Hyper prep kit and the Kapa dual-indexed adapter (KK8722) (Roche) as per the manufacturer’s instructions. Thereafter, a size selection cleanup was done using 0.8× AMPure XP beads (Beckman Coulter, Inc.) to select for amplicons with sequencing adapters and eliminate free adapters, primers, nucleotides, salts and DNA fragments not ligated to adapters. The adapter-ligated libraries were then amplified using Illumina primers and cleaned with 0.8× AMPure beads to select for fragments >450 bp in size. The libraries were quantified using a Qubit dsDNA HS kit on a Qubit fluorometer V3 (Invitrogen), and sizes were verified by the DNA 1000 assay kit using the 2100 Bioanalyzer (Agilent). The libraries were mixed in equimolar concentrations, denatured, spiked with 8% PhiX DNA, and finally sequenced using a MiSeq reagent kit v3 (Illumina) with an output of 2 × 300-bp paired reads.

### Sequence data analysis.

SeekDeep v3.0.1 (65) initially demultiplexed the sequences, based on the MIDs. The paired consensus reads were trimmed and clustered to estimate the frequency of DNA clusters (referred to here as haplotypes). Haplotypes were discarded if they did not occur in duplicate samples and if their combined relative frequency was <1%. However, for *mdr1* (samples from sequencing run 1), samples with and without a replicate were included in the analysis, since the inclusion of the samples without duplicates did not impact the number of unique haplotypes identified. A conservative cutoff of 1% was set based on the lowest (0.5:0.59:0.01; i.e. 50% 3D7, 49% HB3, 1% 7G8) lab isolate mixture, unless the haplotype was independently detected in other samples at >1%. Chimeric reads were considered PCR artifacts and discarded. The SNP and haplotype frequencies in the population were calculated as the number of samples that contained the SNP or haplotype over the total number of samples genotyped. Statistical analysis to generate SNP and haplotype frequency tables was carried out in R v4.0.3 (66).

### *Pfcrt* capillary sequencing and analysis.

The MID tagged *Pfcrt* (PF3D7_0709000) primers failed to generate nested PCR amplicons; therefore, *Pfcrt* was amplified and sequenced using previously described primers and PCR conditions (31, 32) on an ABI3730xl system (Applied Biosystems). The *Pfcrt* sequence assembly was performed in CLC Main Workbench v7.9.1 (Qiagen, UK), and SNPs were identified and called based on the 3D7 reference sequences. Nucleotide positions that displayed a peak within a peak in the sequence chromatograms were noted as “mixed.” Consensus sequences were extracted from the sequence assemblies using CLC Genomics Workbench v9.5.3 and used to construct multiple-sequence alignments in Clustal Omega v1.2.1 (67, 68) to identify SNPs.

### Data availability.

The amplicon sequence data have been deposited in GenBank under the following accession numbers: for *Pfdhfr*, OM370904 to OM370913; for *Pfdhps*, OM370914 to OM370917; for *Pfk13*, OM370918 to OM370923; and for *Pfmdr*, OM370924 to OM370928. The *Pfcrt* sequence data have been deposited in GenBank under the following accession numbers: for codon 76, OM417816 to OM418014, and for codon 145, OM418015 to OM418379.
