# Supplementary material for: Targeted Amplicon Deep Sequencing for Monitoring Antimalarial Resistance Markers in Western Kenya
Source: Antimicrob Agents Chemother. 2022 Mar 10;66(4):e01945-21. doi: 10.1128/aac.01945-21 (PMC9017353; doi:10.1128/aac.01945-21)
Supplement: SUPPLEMENTAL FILE 1 — Tables S1 and S2. Download aac.01945-21-s0001.pdf, PDF file, 0.2 MB [file aac.01945-21-s0001.pdf]

**Supplementary table 1*:*** RT-PCR and nested PCR primer sequences

| Gene | Primer | Sequence 5'-3' | Bases | Reference |
|---|---|---|---|---|
| *18s RNA* | 18s Pf F | GTAATTGGAATGATAGGAATTTACAAGGT | 29 | Hermsen et al.2001 |
| | 18s Pf R | TCAACTACGAACGTTTTAACTGCAAC | 26 | |
| | 18s Pf probe | AACAATTGGAGGGCAAG | 17 | |
| *dhps* | dhps_extF | CTAAACGTGCTGTTCAAAGAATG | 23 | Designed in this study |
| | dhps_extR | GTGGATACTCATCATATACATG | 22 | |
| | dhps_intF | [MID tag]-GATAAATGAAGGTGCTAGTGT | 21 | |
| | dhps_intR | GAGTTTAATAGATTGATCATG | 21 | |
| *k13* | k13_extF | CTTAACTTCTTAAGAAATCCG | 21 | Designed in this study |
| | k13_extR | AGGCATATGGAAATTGTTCCC | 21 | |
| | k13_intF | [MID tag]-GAAGCCTTGTTGAAAGAAGCAG | 22 | |
| | k13_intR | CACCATTAGTTCCACCAATGAC | 22 | |
| *dhfr* | dhfr_extF | CTCCTTTTTATGATGGAACAAGT | 23 | Designed in this study |
| | dhfr_extR | CTTTTCTAAAAATTCTTGATAAAC | 24 | |
| | dhfr_intF | [MID tag]-GAACAAGTCTGCGACGTTTTC | 21 | |
| | dhfr_intR | CTTGATAAACAACGGAACCTCC | 22 | |
| *mdr1* | mdr1_extF | GAGTTGAACAAAAAGAGTACCG | 22 | Designed in this study |
| | mdr1_extR | CCATTAAAGCCTCTTCTATAATGG | 24 | |
| | mdr1_intF | [MID tag]-GTATGTGCTGTATTATCAGG | 20 | |
| | mdr1_intR | GTATTGTTATTATATAACAAAG | 22 | |
| *crt* | crt76_F | GGTGGAGGTTCTTGTCTTGG | 20 | Okombo et al. 2014 |
| | Crt76_R | ATAAAGTTGTGAGTTTCGGATG | 22 | |
| | crt145_extF | CTTATGACCTTTTTAGGAACG | 21 | Designed in this study |
| | crt145_extR | CTTAATATTAAAAAGCAGAAGAAC | 24 | |
| | crt145_intF | AGGAACGACACCGAAGCT | 18 | |
| | crt145_intR | GCAGAAGAACATATTAATAGG | 21 | |
| *ama1* | ama1_ext_F | GGAGAAGATGCTGAAGTAGCTGG | 23 | Designed in this study |
| | ama1_ext_R | GGTATATCTTCACAATTTCCATCG | 24 | |
| | ama1_int_F | [MID tag]-GAAATGTCCAGTATTTGGTAAAGG | 24 | |
| | ama1_int_R | CCCATAATCCGAATTTTGCATTC | 23 | |

**Supplementary Table 2:** Multiplex identifier (MID) 10bp sequence sets

| MID tag | sequence | MID tag | sequence |
|---|---|---|---|
| MID-1 | ACGAGTGCGT | MID-9 | TCTCTATGCG |
| MID-2 | ACGCTCGACA | MID-10 | TGATACGTCT |
| MID-3 | AGACGCACTC | MID-11 | CATAGTAGTG |
| MID-4 | AGCACTGTAG | MID-12 | CGAGAGATAC |
| MID-5 | ATCAGACACG | MID-13 | ATACGACGTA |
| MID-6 | ATATCGCGAG | MID-14 | TCACGTACTA |
| MID-7 | CGTGTCTCTA | MID-15 | CGTCTAGTAC |
| MID-8 | CTCGCGTGTC | MID-16 | TCTACGTAGC |

Amplicons were generated using forward primers tagged on the 5' end with the above listed MIDs. This enabled the unique identification of sequences from each sample during sequence data demultiplexing.

## References

Okombo J, Kamau AW, Marsh K, Sutherland CJ, Ochola-Oyier LI. Temporal trends in prevalence of Plasmodium falciparum drug resistance alleles over two decades of changing antimalarial policy in coastal Kenya. Int J Parasitol Drugs Drug Resist. 2014. 4(3):152-63.

Hermsen CC, Telgt DSC, Linders EHP, Van De Locht LATF, Eling WMC, Mensink EJBM, et al. Detection of Plasmodium falciparum malaria parasites in vivo by real-time quantitative PCR. Mol Biochem Parasitol. 2001. 118(2):247–51.
